# Pre-stroke Disability and Long-Term Functional Limitations in Stroke Survivors: Findings From More of 12 Years of Follow-Up Across Three International Surveys of Aging

**DOI:** 10.3389/fneur.2022.888119

**Published:** 2022-06-14

**Authors:** Andres Gil-Salcedo, Aline Dugravot, Aurore Fayosse, Benjamin Landré, Louis Jacob, Mikaela Bloomberg, Séverine Sabia, Alexis Schnitzler

**Affiliations:** ^1^Université Paris-Cité, Inserm U1153, Epidemiology of Ageing and Neurodegenerative Diseases, Paris, France; ^2^Faculty of Medicine, University of Versailles Saint-Quentin-en-Yvelines, Montigny-le-Bretonneux, France; ^3^Research and Development Unit, Parc Sanitari Sant Joan de Déu, CIBERSAM, Barcelona, Spain; ^4^Department of Epidemiology and Public Health, University College London, London, United Kingdom; ^5^Université Versailles Saint Quentin en Yvelines, EA 4047 Handi-Resp, Service de neurologie hôpital A. Mignot, Garches, France

**Keywords:** stroke, chronic phase, premorbid disability, functioning, limitation

## Abstract

**Background:**

Almost 50% of the post-stroke disabled population already have a premorbid disability before stroke. These patients may be offered a different care pathway in the acute and subacute phase than those without pre-morbid disability. Therefore, the aim of this study was to assess the association of the severity of premorbid disability with change of limitations in basic and instrumental activities of daily living (ADL/IADL) 1 year after stroke and over the following decade.

**Methods:**

Among 3,432 participants from HRS, SHARE and ELSA cohorts with a first stroke, ADL/IADL limitations were measured at 1–2 years prior to stroke, at 1 year post-stroke, and during the chronic phase. Modified Ranking Scale (P-mRS) was used to categorize the participants by level of premorbid disability (1–2 years pre-stroke). Change in ADL/IADL limitations by P-mRS level (0–1, 2–3, and 4–5) was assessed using a piecewise linear mixed model with a breakpoint set at 1 year post-stroke, stratified by median age groups.

**Results:**

Increase in ADL limitations at 1 year post-stroke was less pronounced in P-mRS ≥2 (*p* < 0.005). After years of relative stability, limitations of ADL increased for all P-mRS levels (*p* = 0.003). In those aged ≥75 years at stroke event, the increase was similar irrespective of P-mRS (*p* = 0.090). There were no significant differences in IADL trajectories between P-mRS levels (*p* ≥ 0.127).

**Conclusion:**

These results suggest similar trajectories of functional limitations between P-mRS levels up to 9 years post-stroke, highlighting the possible benefit of including patients with pre-morbid disability to certain treatments during the acute phase.

## Introduction

Stroke is one of the leading causes of long-term disability in the world (1, 2). More than 20% of stroke survivors experience some limitations in activities of daily living (ADL) (3, 4) and 30% experience limitations in instrumental activities of daily living (IADL) (4, 5), with prevalence of limitations increasing with age (6). As populations age (1, 4) and stroke events occur at more advanced ages, some patients have functional limitations before stroke event (premorbid disability). The care pathway for these patients differs during the acute and sub-acute phase compared to those without premorbid disability. In international reports (7, 8) and a previous study (9) it was reported that only 1 out of 10 patients treated with intravenous thrombolysis had a premorbid disability and in one study (10) only 3 out of 10 patients with premorbid disability accessed a full rehabilitation service during acute care following stroke, despite lack of evidence to support such differential treatment (10, 11). These disparities in the care pathway between those with and without premorbid disability might contribute to increase health inequalities between these groups (11, 12).

Long term studies have reported that about 50% of patients with disability after stroke had a premorbid disability (13–15). Studies suggested after stroke, persons with premorbid disability have higher rates of mortality, institutionalization, and healthcare costs (13, 14), higher severity ratios (14, 16) and a lower probability of achieving favorable outcomes (9) compared to those without premorbid disability. As a result, healthcare professionals tend to develop cognitive biases during acute care of patients with premorbid disability (11, 14). In particular, *fragility* refers to the tendency of physicians to assign pessimistic prognoses to patients with premorbid disability (11, 17) and therefore withhold certain treatments.

Most long-term studies so far [with a maximum of 10 years of follow-up in one study (18)] use the premorbid modified Rankin scale (P-mRS) in dichotomous form) (no-disability [levels 0–1 or 0–3] vs. disability [levels >2 or >3]) (14, 15). Although the P-mRS has been shown to be a predictor of prognosis in its dichotomous form (15, 19), this scale could provide additional information on the influence of the severity of premorbid disability on the functional outcome of stroke patients if a broader categorization (no disability, mild or moderate disability, and severe disability) was used. In addition, there is no evidence of its impact on change in a more graded disability scale, such as limitations in ADL and IADL (20), following stroke event while differentiating the post-stroke subacute and chronic phases (21).

In order to address these gaps in the literature, we examined the association between premorbid disability severity and ADL/IADL limitations after stroke both 1 year after stroke (after the subacute phase) and in the long term, using data spanning 20 years from three large-scale cohort studies conducted in Europe and the United States.

## Methods

### Study Population

The study population was drawn from three related surveys (22) of persons aged over 50 from the United States and Europe: the Health and Retirement Study (HRS) (23), the Survey of Health, Aging and Retirement in Europe (SHARE) (24) and the English Longitudinal Study of Ageing (ELSA) (25). Details of these studies are provided elsewhere (23–25). For the present study, HRS data from 1996 to 2018 (12 waves), SHARE data from 2004 to 2016 (6 waves; no data in 2008), and ELSA data from 2002 to 2018 (9 waves) were used. The study sample included all participants who reported having been diagnosed by a physician as having suffered a stroke from the three studies and with data on limitations and covariates before stroke onset (1–2 years pre-stroke) and at least one wave post-stroke. Participants who reported a prevalent stroke at baseline were excluded because no data prior to stroke were available. Participants with recurrent stroke have different prognosis and care than individuals with a single stroke event (26, 27), and were therefore excluded.

### ADL and IADL Limitations and Follow-Up

ADL and IADL data were collected similarly in the three surveys (28). Participants (or proxies) reported whether they had experienced any difficulty with ADLs or IADLs lasting longer than 3 months due to a “physical, mental, emotional or memory problem.” ADLs included dressing, walking across a room, bathing/showering, eating, getting in/out of bed, using the toilet, and urinary continence, leading to an ADL score ranging from 0 to 7 (29). IADLs included using a map, preparing a hot meal, shopping for groceries, using the telephone, taking medications, and managing money, leading to an IADL score ranging from 0 to 6 (30). Scores equal to 0 indicated no limitations and a score of 7 (for ADLs) or 6 (for IADLs) indicated respondents were fully limited. Follow-up of participants started at year of premorbid stroke status (1 or 2 years pre-stroke) and ended at the last wave of ADL/IADL data, using all information available at waves in between. ADL/IADL limitations show important short-term changes during the acute phase (first year post-stroke) (31) and given that data were collected every 2 years, data in the year following stroke were not considered in the analysis.

### Premorbid Disability Level

Premorbid disability level was evaluated based on the P-mRS using self-reported limitations at the last wave before stroke, allowing a maximum of 2 years before stroke event. The P-mRS is a scale for determining levels of disability prior to stroke and has been shown to be a strong predictor of prognosis (19). Three levels of P-mRS were estimated using ADLs and IADLs at the last wave before stroke: 0–1 (no symptoms or significant disability: no limitations in ADLs or IADLs); 2–3 (slight-to-moderate disability: some limitations in ADLs and/or IADLs but able to walk across a room); and 4–5 (moderately severe and severe disability: multiple limitations in ADLs and/or IADLs including an inability to walk across room) (32, 33).

### Covariates

Sociodemographic factors included sex, age, education (below secondary, secondary, and above secondary level based on a previously harmonized education category) (34) and marital status (“married or cohabiting” vs. “single, divorced or widowed”) and were drawn from the closest wave before stroke event. Other covariates were drawn concurrently with measures of ADL/IADL limitations at each wave. Health behaviors included smoking status (non-smoking and current smoking), alcohol consumption over the last 6 months [abstainers (<once a month), moderate drinkers (≥once per month to <5 days/week) and frequent drinkers (≥5days/week)], and practice of moderate-to-vigorous physical activity at least three times a week. Body mass index (BMI) was estimated based on self-reported weight and height and categorized as <18.5–25, 25–30, and >30 kg/m^2^. In ELSA information on BMI was available every 2 waves and data were carried forward for missing waves. Morbidities included self-report of medical diagnosis of heart problems, high blood pressure, diabetes, lung diseases, arthritis, cancer, chronic pain, and sleep disorders. The number of morbidities was categorized as 0, 1, 2, or ≥3.

### Statistical Analysis

Characteristics of the population as a function of P-mRS level (0–1, 2–3, and 4–5) at the closest interview before stroke (pre-stroke status) were presented. Pearson's chi-squared test was used to assess differences between groups in sociodemographic factors, health behaviors, BMI categories, and the number of morbidities. For continuous scores of ADL/IADL limitations, analysis of variance was used to describe differences by P-mRS level groups. In addition, a multivariate logistic model was used to compare participants with “no symptoms or significant disability” (P-mRS 0–1) vs. premorbid disability (P-mRS ≥2), and stratified by age group (50–74 and ≥75 years) defined using median age.

#### Main Analysis

Change in ADL/IADL limitation scores, at 1 year post-stroke and in the long-term, was assessed using piecewise linear mixed models. The origin of the timeline in the analysis was the year of stroke. The start of the timeline was set at 1 year before stroke using data from the last wave before stroke (allowing a maximum of 2 years before stroke event). The next measure included in the analysis was the first in the data assessed after 1 year post-stroke (after subacute phase). The breakpoint in the model was set at 1 year post-stroke to examine change in limitations following the subacute phase. Random effects were included for intercept at survey-level (HRS, SHARE, and ELSA) and intercept and slope at the individual level to account for variations between surveys and individuals. Analysis were adjust sequentially, initially including time, P-mRS level, sociodemographic variables, and year of stroke. Time squared and lower order interactions between covariates and time terms (time, time squared) were included in the model if they were significant based on the likelihood ratio test. The model was then further adjusted for health behaviors, BMI and number of morbidities as time-dependent variables.

A significant interaction was found between age of stroke as a continuous term and P-mRS both for ADL [both at the intercept (*p* < 0.001) and over time (*p* = 0.001)] and IADL limitations (intercept *p* < 0.001). Exploratory analysis stratified in 6 age bands (Supplementary Figure 1) led us to stratify the analysis into two age groups (individuals aged 50–74 years at stroke year and those aged ≥75 years) using the median age of the sample to ensure sufficient numbers in each group. No further interaction was found within these age groups.

To facilitate interpretation of the results, we plotted the adjusted mean ADL/IADL limitation score as a function of time from stroke event estimated in fully adjusted models with 95% confidence intervals (95%CI). These results were plotted over a period of 16 years for the age group 50–74 years, and 12 years for age group ≥75 years, representing the maximum period for which the number of participants in each level of P-mRS was at least 5.

All statistical analyses were carried out using STATA statistical software version 15.1 (Stata-Corp, College Station, Texas).

### Sensitivity Analysis

We undertook several sensitivity analyses to test the robustness of our results. First, analyses were carried out separately in HRS, SHARE, and ELSA to evaluate the influence of each survey. Second, imputation of ADL/ADL limitations at 1 year before stroke was performed for participants with limitation data only at 2 years before stroke. We imputed values based on predictions from a fully adjusted linear mixed model with data from 1 and 2 years before stroke, and then repeated the main analysis to evaluate whether limitations increased during the year before stroke where limitation data were missing. Third, because the follow-up period differed between surveys [means (range): HRS = 5.7 (0–21), SHARE = 3.4 (0–11), ELSA = 5.8 (0–16)], the analyses were repeated restricting the follow-up to 6 years (median of total follow-up) to evaluate the influence of follow-up periods of different lengths. Finally, premorbid disability was associated with a high risk of mortality after stroke, potentially impacting estimates of change in limitations in stroke survivors; thus, we replicated the analysis excluding participants who died during follow-up to assess the effect of mortality on results.

## Results

### Population Characteristics

**Figure 1:**
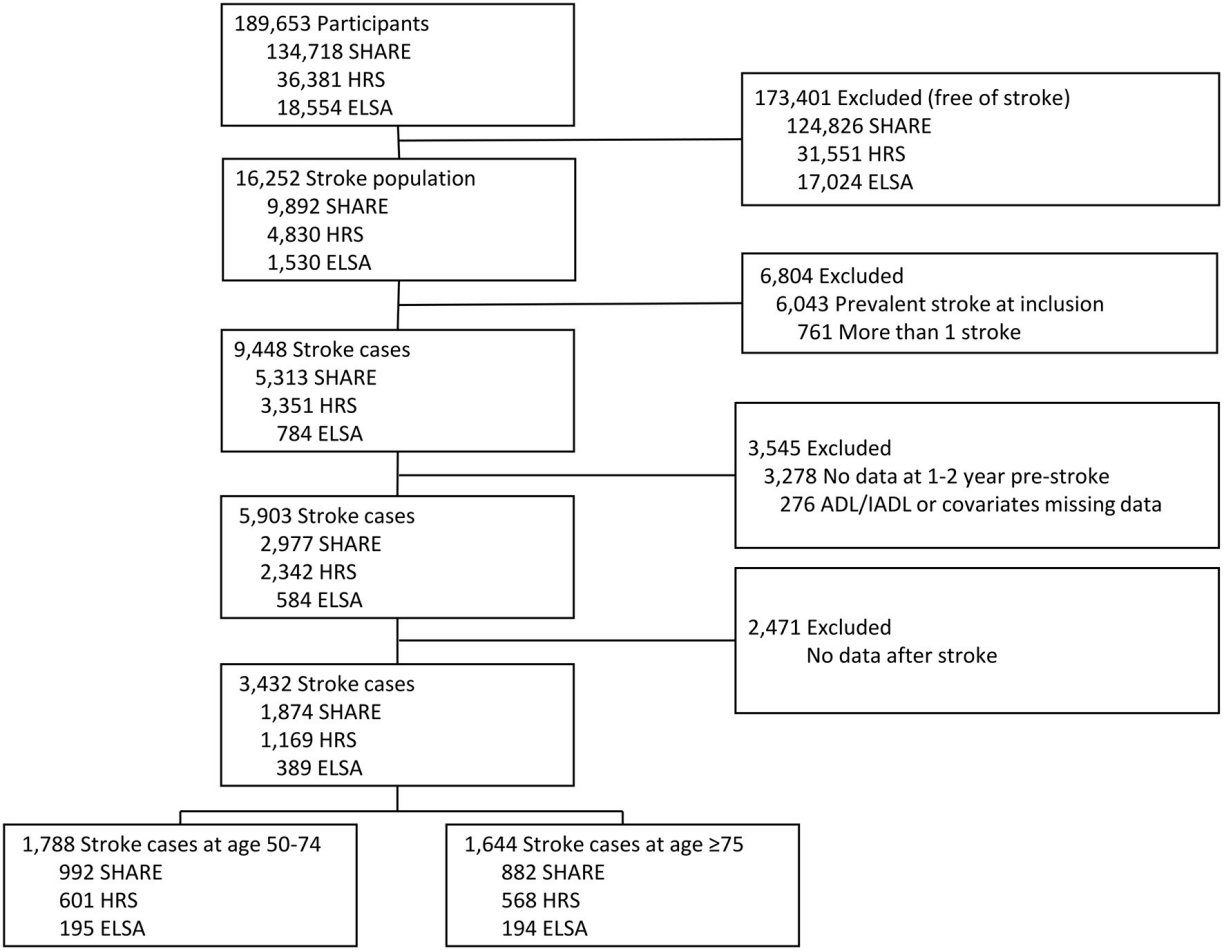
Flowchart of the study samples.

Of the 189,653 participants from the 3 surveys, 16,252 individuals reported a history of stroke. 6,804 participants were excluded due to prevalent stroke at study baseline (*N* = 6,043) or multi-stroke event over the follow-up period (*N* = 761). Of 9,448 participants with first-ever stroke, 3,545 did not participate 1–2 years pre-stroke or had missing ADL/IADL limitation or covariate data at this wave. Finally, 2,471 participants were excluded due to non-participation following the subacute phase, leading to an analytic sample of 3,432 first-ever stroke cases (1,169 in HRS; 1,874 in SHARE; 389 in ELSA) (Figure 1).

At the last interview before stroke (1,541 1 year before stroke and 1,891 2 years before stroke), the mean age of participants was 73.5 (SD = 9.7) years, 55% were female, 54% reported no limitation, 27% reported slight-to-moderate disability (P-mRS 2–3), and 18% reported moderately-severe and severe disability (P-mRS 4–5). Participants in the slight moderate (P-mRS 2–3) and moderate severe and severe (P-mRS 4–5) disability groups compared to those with no limitations (P-mRS 0–1), were older, more likely to be women, with lower educational level, single/divorced/widowed, non-smokers, non-drinkers, obese (BMI ≥ 30 kg/m^2^) and less likely to take part in moderate and vigorous physical activities; they also tended to have a higher number of comorbidities (all *p* < 0.001; Table 1). These differences were evident in both age groups (Supplementary Tables 1, 2).

**Table 1:** Characteristics of the study sample at last^a^ interview before stroke onset according to premorbid disability level using modified Rankin scale (P-mRS)^b^.

	**Premorbid Disability Level**			
**Characteristics**	**P-mRS 0–1**	**P-mRS 2–3**	**P-mRS 4–5**	***p***
	**(*N =* 1,872)**	**(*N =* 925)**	**(*N =* 635)**	
**Sex**
Men	984 (52.6)	344 (37.2)	215 (33.9)	<0.001
Women	888 (47.4)	581 (62.8)	420 (66.1)	
**Age (years)**
50–65	547 (29.2)	220 (23.8)	137 (21.6)	<0.001
66–74	553 (29.5)	211 (22.8)	120 (18.9)	
75–81	457 (24.4)	232 (25.1)	161 (25.4)	
>81	315 (16.8)	262 (28.3)	217 (34.2)	
**Education level**
Low	543 (29.0)	366 (39.6)	317 (49.9)	<0.001
Middle	917 (49.0)	415 (44.9)	241 (38.0)	
High	412 (22.0)	144 (15.6)	77 (12.1)	
**Marital status**
Single/divorced/widowed	695 (37.1)	412 (44.5)	349 (55.0)	<0.001
Married/Cohabiting	1,177 (62.9)	513 (55.5)	286 (45.0)	
**Smoking status**
Non-smoking	889 (47.5)	578 (62.5)	404 (63.6)	<0.001
Current smoking	983 (52.5)	347 (37.5)	231 (36.4)	
**Alcohol consumption**
Non-drinkers	900 (48.1)	586 (63.4)	506 (79.7)	<0.001
Moderate drinkers	636 (34.0)	208 (22.5)	69 (10.9)	
Heavy drinkers	336 (18.0)	131 (14.2)	60 (9.5)	
**MVPA at least 3 times a week**
No	700 (37.4)	564 (61.0)	544 (85.7)	<0.001
Yes	1,172 (62.6)	361 (39.0)	91 (14.3)	
**BMI (kg/m** ^2^ **)**
<25	608 (32.5)	289 (31.2)	167 (26.3)	
25–30	779 (41.6)	354 (38.3)	215 (33.9)	
>30	485 (25.9)	282 (30.5)	253 (39.8)	
**Number of comorbidities**
0	322 (17.2)	50 (5.4)	25 (3.9)	<0.001
1	567 (30.3)	166 (18.0)	66 (10.4)	
2	449 (24.0)	243 (26.3)	113 (17.8)	
3 or more	534 (28.5)	466 (50.4)	431 (67.9)	
**ADL limitation Score (0–7)** ^**c**^
Mean (SD)	0.0 (0.0)	1.0 (0.9)	2.8 (1.8)	<0.001
**IADL limitation Score (0–6)** ^**c**^
Mean (SD)	0.0 (0.0)	1.0 (1.2)	1.7 (1.9)	<0.001

*^a^Interview maximum 2 years before stroke*.

*^b^Values are numbers (percentages)*.

*^c^Score corresponds to number of ADL or IADL limitations. Percentages are reported in column. ADL, Activities of daily living; IADL, Instrumental ADL; P-mRS, premorbid modified Ranking Scale; MVPA, moderate-to-vigorous physical activity*.

In the multivariate logistic model comparing participants with no premorbid disability (P-mRS 0–1) to those with premorbid disability (P-mRS ≥2, 38% for those aged 50–74 and 53% for those aged ≥75), participants who were women, with lower educational level and more comorbidities were more likely to have a premorbid disability (all *p* < 0.05) in both age groups. Age and obesity were associated with higher odds of premorbid disability only for participants aged ≥75, and single/divorced/widowed participants were more likely to have disability only in those aged 50–74 (Supplementary Table 3).

### Change in the Number of ADL Limitations

**Figure 2:**
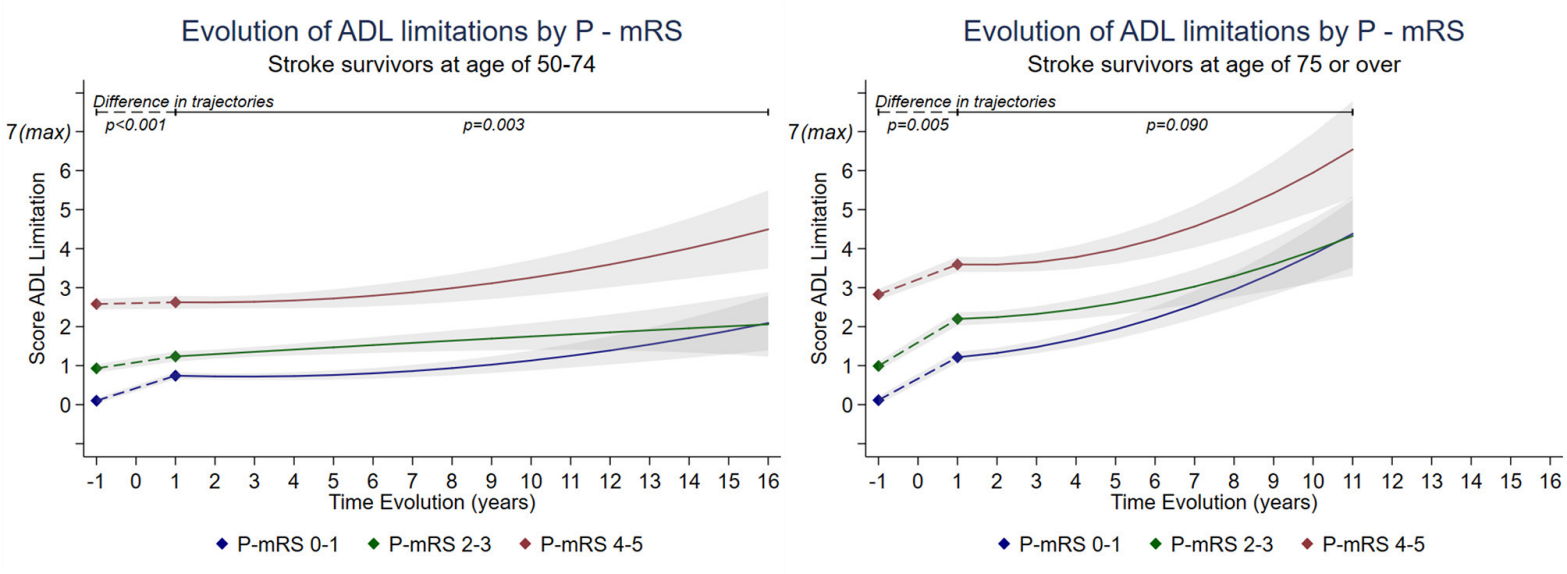
Long-term trajectories of score of ADL limitations in stroke survivors by premorbid modified Rankin scale (P-mRS) stratified by age*. *Estimated from piecewise linear mixed model adjusted for socioeconomic variables, pre-stroke wave data collection, heath behaviors, BMI, and number of comorbidities. Breakpoint was set at 1 year post-stroke. Detailed changes in limitations between pre-stroke and 1 year post-stroke could not be modeled due to lack of information and was assumed linear for the sake of the analysis. ADL score range from 0 = no limitation to 7 = maximum limitation. Estimations are presented for a 16 year follow-up (corresponding to maximum follow-up with at least 5 participants by level of mRS) for population aged 50–74 years and a 11 year follow-up for those aged ≥75 years. Supplementary Table 4.

The mean follow-up was 5.1 (SD = 3.9) years for all levels of premorbid disability in the group aged 50–74 years and 3.6 (SD = 2.7) years in the group aged ≥75 years. The change in the number of ADL limitations between pre-stroke and 1 year post-stroke differed as a function of premorbid disability levels (*p* < 0.005). At 1 year post-stroke, participants aged 50–74 years with no premorbid limitations (P-mRS 0–1) showed a 9% increase (ΔADL = 0.64, 95%CI = 0.54–0.75, out of a maximum of 7 limitations) in ADL limitations compared to their pre-stroke ADL limitations score (Figure 2, Supplementary Table 4). This increase was 4% among those with slight-to-moderate premorbid limitation (P-mRS 2–3; ΔADL = 0.30, 95%CI = 0.15–0.46) and 0.6% among those with premorbid moderately severe to severe limitation levels (P-mRS 4–5; ΔADL = 0.04, 95%CI = −0.14–0.24; Figure 2, Supplementary Table 4). Among participants aged ≥75 years at stroke event, individuals with no limitation or slight-to-moderate premorbid disability levels had a similar increase (15.7% [ΔADL = 1.10 95%CI = 0.94–1.26] and 17.2% [ΔADL =1.21 95%CI = 1.02–1.39] respectively; *p* = 0.388) in ADL limitations pre-stroke and 1 year post stroke while those with moderate-to-severe premorbid disability tended to experience a lower increase in ADL limitations (11%[ΔADL = 0.77 95%CI = 0.56–0.98], *p* < 0.001).

When examining the long-term trajectories of ADL limitations (after 1 year post-stroke), in participants aged 50–74 at stroke event, only P-mRS 2–3 showed a significant 5.0% increase (ΔADL =0.35 95%CI = 0.11–5.85, *p* = 0.004) between years 1 and 7. Then, between 7 and 16 years post-stroke, those with premorbid disability level 0–1 and 4–5 showed a significant increase of 17.0% (ΔADL =1.23 95%CI = 0.59–1.86, *p* < 0.001) and 23.0% in ADL limitations score respectively (ΔADL =1.61 95%CI = 0.76–2.46, *p* < 0.001), while those with premorbid disability level of 2–3 showed no significant increase. In participants aged ≥75 years at stroke event, there were no significant differences in ADL change 1 year post-stroke between premorbid disability levels (*p* = 0.090, Figure 2, Supplementary Table 4). Between post-stroke years 1 and 4, individuals with p-mRS levels at 0–1 and 2–3 had a mean increase in ADL score of 6.6% (ΔADL = 0.46 95%CI = 0.26–0.66, *p* < 0.001) and 3.5% (ΔADL = 0.25 95%CI = 0.01–0.49, *p* = 0.050), respectively, while those with level 4–5 had a non-significant increase of 2.7% (ΔADL = 0.19 95%CI = −0.11 to 0.48, *p* = 0.211). Between 4 and 12 years post-stroke, all levels of P-mRS showed a similar increase in ADL limitations: 46.7% (ΔADL =3.27 95%CI = 2.26–4.28, *p* < 0.001) for level 0–1, 32.8% (ΔADL = 2.29 95%CI = 1.11–3.48, *p* < 0.001) for level 2–3 and 48.8% (ΔADL = 3.42 95%CI = 1.99–4.84, *p* < 0.001) for level 4–5.

### Change in the Number of IADL Limitations

**Figure 3:**
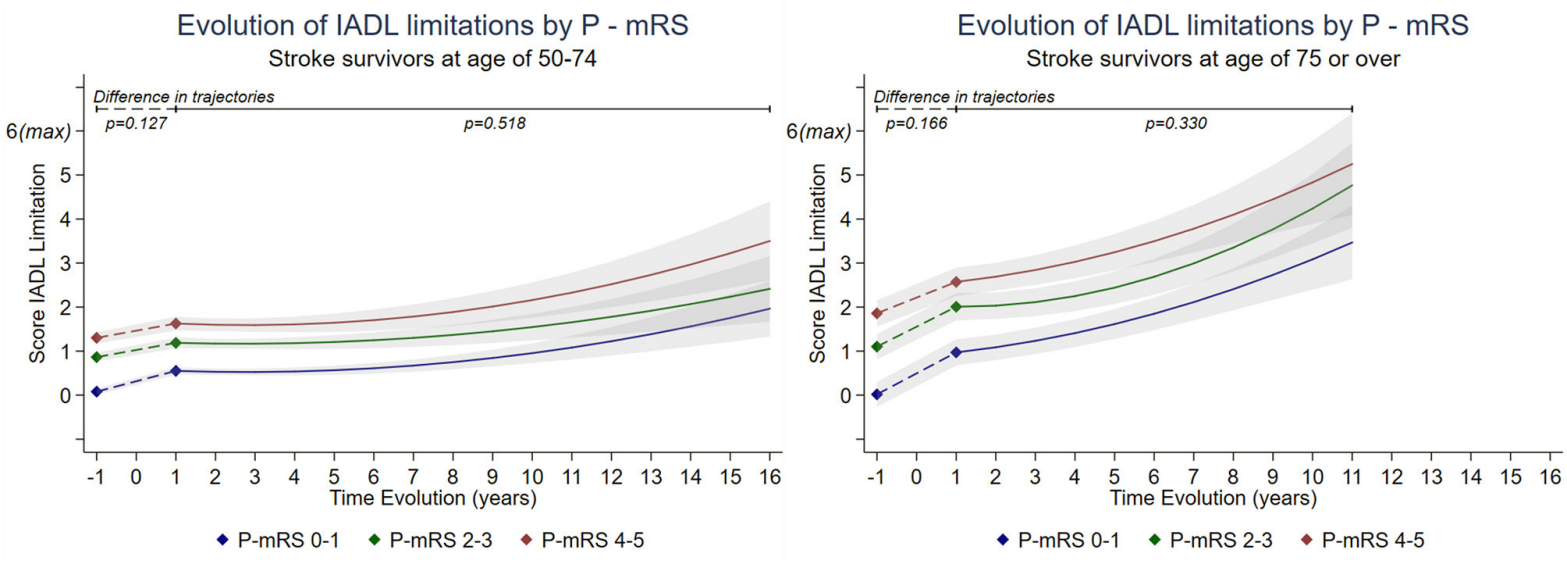
Long-term trajectories of score of IADL limitations in stroke survivors by premorbid modified Rankin scale (P-mRS) stratified by age*. *Estimated from piecewise linear mixed model adjusted for socioeconomic variables, pre-stroke wave data collection, heath behaviors, BMI, and number of comorbidities. Breakpoint was set at 1 year post-stroke. Detailed changes in limitations between pre-stroke and 1 year post-stroke could not be modeled due to lack of information and was assumed linear for the sake of the analysis. IADL score range from 0 = no limitation to 6 = maximum limitation. Estimations are presented for a 16 year follow-up (corresponding to maximum follow-up with at least 5 participants by level of mRS) for population aged 50–74 years and a 11 year follow-up for those aged ≥75 years. Supplementary Table 5.

The trajectories of IADL limitations over the follow-up did not differ by premorbid disability level for both age groups (Figure 3, Supplementary Table 5). Among participants aged 50–74 years at stroke event, at 1 year post-stroke, increase in IADL limitation score was similar whether the premorbid disability level was 0–1 (7.9% increase, ΔIADL = 0.47 95%CI = 0.37–0.57 out of a maximum of 6 limitations), 2–3 (5.4%, ΔIADL = 0.32 95%CI = 0.19–0.46), or 4–5 (5.4%, ΔIADL = 0.32 95%CI = 0.15–0.50; comparison ΔIADL for 0–1 vs. 2–3, *p* = 0.07; for 0–1 vs. 4–5, *p* = 0.146). Changes of IADL limitations did not differ by premorbid disability levels for those aged ≥75 at 1 year post-stroke (*p* = 0.166). The increase in IADL score was 15.1% (ΔIADL = 0.95 95%CI = 0.80–1.10) for level 0–1, 15.8% (ΔIADL = 0.90 95%CI = 0.72–1.08) for level 2–3 and 11.9% (ΔIADL = 0.71 95%CI = 0.51–0.91) for level 4–5.

Long-term trajectories showed no significant differences between levels of premorbid disability. Between post-stroke years 1 and 7 in participants aged 50–74 years, there was no evidence of change in ADL limitations irrespective of premorbid disability level (ΔIADL <3% and p>0.115 for all levels). A significant increase in IADL limitations was then observed between years 7 and 16 post-stroke in all levels: 21.0% (ΔIADL = 1.29 95%CI = 0.71–1.87, *p* < 0.001) for level 0–1, 18.6% (ΔIADL = 1.11 95%CI = 0.45–1.78, *p* = 0.001) for level 2–3, and 28.6% (ΔIADL = 1.72 95%CI = 0.94–2.49, *p* < 0.001) for level 4–5. In participants aged ≥75 years, the increase of IADL limitations was observed from 1 year post-stroke; level 0–1 and 4–5 showed an increase of 7.0% (ΔIADL = 0.44 95%CI = 0.25–0.62, *p* = 0.001; ΔIADL = 0.45 95%CI = 0.18–0.73, *p* = 0.001 respectively) and level 2–3 showed an increase of 4.0% (ΔIADL = 0.24 95%CI = 0.13–0.47, *p* = 0.001) during a follow-up between year 1 and 4 post-stroke. Then, between years 4 and 12 post-stroke a more pronounced increase was observed irrespective of premorbid disability levels; 41.2% (ΔIADL = 2.48 95%CI = 1.53–3.41, *p* < 0.001) for those in level 0–1, 51.6% (ΔIADL = 3.10 95%CI = 2.00–4.20, *p* = 0.001) for those in level 2–3, and 44.5% (ΔIADL = 2.67 95%CI = 1.35–3.99, *p* = 0.001) for those in level 4–5.

### Sensitivity Analysis

In the analyses stratified by survey, the results at 1 year post-stroke showed similar findings as the main analysis. Long-term trajectories were found to be similar to the main results, except for ADL changes in SHARE and IADL in ELSA, possibly due to absence of data with long follow-up (Supplementary Figures 2, 3). Second, in analyses with imputation of ADL/IADL limitations score at 1 year pre-stroke, the results were similar to the main analysis suggesting that possible increase in limitations between 2 and 1 years pre-stroke did not influence the change in limitations during post-stroke follow-up (Supplementary Figure 4). Third, analyses repeated with period of follow-up limited to 6 years post-stroke showed similar results to the main analysis suggesting that results were not influenced by the length of follow-up (Supplementary Figure 5). Finally, removal from analysis of participants who died during the follow-up period (P-mRS 0–1 = 213, 2–3 = 200, 4–5 = 198, Supplementary Table 6) did not alter findings, suggesting mortality did not influence findings (Supplementary Figure 6).

## Discussion

In this observational study of 3,432 stroke participants with a premorbid disability measure drawn from three international longitudinal surveys, premorbid disability was observed in around 45% of participants. The increase of ADL limitations at 1 year post-stroke was less pronounced in cases with premorbid disability (P-mRS levels 2–5 for those aged 50–74 years and 4–5 for those aged ≥75 years) compared with those reporting none. In the long term, relative stability in ADL limitations was observed between 1 and 7 year post-stroke for those aged 50–74 years and between 1 and 4 years for those aged ≥75 years. Thereafter, a similar increase was observed irrespective of the premorbid disability level, with the exception of those aged 50–74 years with moderate to severe prémorbide disability (P-mRS levels 4–5) where the increase was slightly more pronounced. The trajectories of IADL limitations did not differ between premorbid disability levels.

### Comparison With Previous Studies

In agreement with previously reported studies with long-term follow-up undertaken in populations with premorbid disability (14, 15, 18), we observed a high proportion of participants reporting limitations prior to stroke, especially older participants. Previous studies showed that long-term outcomes in participants with premorbid disability tended to be less favorable with age (13–15, 35, 36); the present study adds to evidence that trajectories of ADL limitations post-stroke differed by premorbid disability levels, particularly among participants with stroke at younger age. In addition, our analyses accounting for pre-stroke ADL limitations allowed us to observe an increase in ADL limitations at 1 year post-stroke that was less pronounced for those with slight to severe premorbid disability (P-mRS 2–5). This finding is consistent with a previous study that showed a higher increase in mRS 3 months post-stroke among individuals with no premorbid limitations (14). Finally, a recent study showed that patients with premorbid disability treated with thrombectomy had no functional differences with patients who did not have premorbid disability 3 months after stroke (37). This contrasts with our results, in which the trajectories of ADL limitations differed according to P-mRS levels at 1 year post-stroke, however this difference ceases to be significant after several years.

### Strengths and Limitations

This observational study has several strengths including a large number of participants with multi-country data on premorbid disability status assessed prospectively before stroke onset. This allowed us to stratify the analysis by age and examine the differences in the trajectories of limitations by levels of premorbid disability maintaining a sufficient number of participants over the follow-up period to ensure robust results. The use of a piece-wise mixed model allowed the examination of two different periods (pre-stroke to 1 year, and 1 to 16 years after stroke) in the same model taking into account the association with both the end of the sub-acute post-stroke phase and long-term trajectory of functional limitations (38).

Our results should be considered in light of the following limitations: (1) subtype of stroke information was not available and stroke was self-reported or reported by a proxy, introducing a possible recall bias. Nevertheless, in previous studies it was observed that prevalence of self-report of chronic conditions such as stroke is close to prevalence obtained from linkage to electronic medical records with agreement of 96% (39), Even so, previous studies show a range of sensitivities between 36 and 98% and a specificity between 96 and 99% (40). Inclusion of false positive (confusion with stroke synonyms) (41) might have affected the trajectory of those with no significant disability (P-mRS 0–1). (2) Data on premorbid status was collected in a period of maximum 2 years before the stroke onset, thus participants measured 2 years pre-stroke may present a different premorbid state at stroke onset compared to participants with measure at 1 year pre-stroke. To assess potential selection bias, an analysis with data imputed for those measured at 2 years pre-stroke was conducted and results were consistent with main findings. (3) Previous studies reported an association between premorbid disability level and mortality, and long-term results may be influenced by the death of participants with a higher level of disability (38, 42). To address this limitation, the analysis was repeated excluding all cases with a report of death during follow-up; the results were consistent with the main findings. (4) We used three different surveys which might increase heterogeneity in the measures; however, previous studies showed good concordance between cohorts and sensitivity analyses stratified by survey suggest that our results were similar across cohorts (28). (5) The P-mRS was derived from the count of ADL and IADL limitations, this scale will require further validation in the premorbid population, although it is beginning to be commonly used to assess the pre-stroke disability level retrospectively in research and clinical settings; the P-mRS was used to categorize the premorbid disability level of stroke participants giving a more clinically significant and comprehensive characterization of premorbid disability than the simple count of ADL and IADL limitations.

### Clinical Implications and Future Research

The clinical care of stroke patients may be different when the patient has a premorbid disability, because of the tendency to establish a pessimistic prognosis for patients with a disability (*fragility* bias) (11, 17). However, our findings suggest that after 1 year post-stroke the increase in limitations is less pronounced for participants with premorbid disability and in the long term all levels showed several years of relative stability. In addition, change in IADL limitations does not differ by severity level of premorbid disability. These findings should be confirmed in future prospective studies, and may encourage healthcare professionals to treat patients with premorbid disability more rigorously during the acute phase of stroke. Our findings suggest the importance of future research aimed at understanding the impact of premorbid disability in acute illness, and addressing possible bias in health care. In addition, future studies should consider trajectories of different types of disability (physical, sensory, or cognitive) after an acute event. Further studies are also needed in low-income countries, where access to preventive and rehabilitative health services may be more restricted.

## Conclusion

The present study may indicate that premorbid disability is present in more than 1/3 of stroke survivors and the trajectories of ADL limitations may be influenced by premorbid disability after several years of relative stability. After the stable period, an increase in ADL limitations is more pronounced in those with severe premorbid disability levels. IADL limitations have a similar trajectories regardless of the level of premorbid disability. These results highlight the importance of adapting health and social care for stroke survivors toward greater inclusion of patients with premorbid disability.

## Data Availability Statement

Publicly available datasets were analyzed in this study. This data can be found here: Health and Retirement Study (HRS), https://hrsdata.isr.umich.edu/data-products, Survey of Health, Aging and Retirement in Europe (SHARE), http://www.share-project.org/ data-access/share-conditions-of-use.html and English Longitudinal Study of Aging (ELSA), https://www.elsa-project.ac.uk/accessing-elsa-data.

## Author Contributions

AG-S and AS: conceptualization, data curation, and writing—original draft preparation. AG-S, SS, AD, and AF: methodology. SS, AD, AF, and AS: validation. AG-S, AD, and AF: formal analysis and visualization. AD, AF, LJ, MB, and BL: writing—review and editing. AS: supervision. All authors contributed to the article and approved the submitted version.

## Funding

This present study was financed by the University of Paris in the framework of pdh's training. The HRS was sponsored by the National Institute on Aging (Grant No. NIA U01AG009740) and was conducted by the University of Michigan. The SHARE data collection has been primarily funded by the European Commission through the FP5 (QLK6-CT-2001-00360), FP6 (SHARE-I3: RII-CT-2006-062193, COMPARE: CIT5-CT-2005-028857, SHARELIFE: CIT4-CT-2006-028812), and FP7 (SHARE-PREP: N 211909, SHARE-LEAP: 227822, SHARE M4: N 261982). Additional funding from the German Ministry of Education and Research, the U.S. National Institute on Aging (U01_AG09740-13S2, P01_AG005842, P01_AG08291, P30_AG12815, R21_AG025169, Y1-AG-4553-01, IAG_BSR06-11, OGHA_04-064), and from various national funding sources is gratefully acknowledged (see www.share-project.org). The ELSA data were made available through the UK Data Archive. ELSA was developed by a team of researchers based at the NatCen Social Research, University College London, and the Institute for Fiscal Studies. The data were collected by NatCen Social Research. The funding was provided by the National Institute of Aging in the United States and a consortium of UK government departments coordinated by the Office for National Statistics. The developers and funders of ELSA and the Archive do not bear any responsibility for the analyses or interpretations presented here.

## Conflict of Interest

The authors declare that the research was conducted in the absence of any commercial or financial relationships that could be construed as a potential conflict of interest.

## Publisher's Note

All claims expressed in this article are solely those of the authors and do not necessarily represent those of their affiliated organizations, or those of the publisher, the editors and the reviewers. Any product that may be evaluated in this article, or claim that may be made by its manufacturer, is not guaranteed or endorsed by the publisher.
