# Supplementary material for: Pre-stroke Disability and Long-Term Functional Limitations in Stroke Survivors: Findings From More of 12 Years of Follow-Up Across Three International Surveys of Aging
Source: Front Neurol. 2022 Jun 14;13:888119. doi: 10.3389/fneur.2022.888119 (PMC9237334; doi:10.3389/fneur.2022.888119)
Supplement: Supplementary file 1 [file Data_Sheet_1.docx]

Supplementary Material

# Table S1. Characteristics of the study sample aged 50-74 at last* interview before stroke onset according premorbid disability level using modified Rankin scale (P-mRs) †.

|  | **Premorbid Disability Level of participants aged 50-74** | | | ***p*** |
| --- | --- | --- | --- | --- |
| **Characteristics** | **P-mRs 0-1** | **P-mRs 2-3** | **P-mRs 4-5** |  |
|  | (N=1,100) | (N= 431) | (N=257) |  |
| Sex |  |  |  |  |
| Men | 594 (54.0) | 179 (41.5) | 106 (41.3) | <0.001 |
| Women | 506 (46.0) | 252 (58.5) | 151 (58.8) |  |
| Age (years) |  |  |  |  |
| 50-64 | 547 (49.7) | 220 (51.0) | 137 (53.3) | 0.587 |
| 65-74 | 553 (50.3) | 211 (49.0) | 120 (46.7) |  |
| Education level |  |  |  |  |
| Low | 286 (26.0) | 162 (37.6) | 111 (43.2) | <0.001 |
| Middle | 566 (51.5) | 203 (47.1) | 110 (42.8) |  |
| High | 248 (22.6) | 66 (15.3) | 36 (14.0) |  |
| Marital status |  |  |  |  |
| Single/divorced/widowed | 348 (31.6) | 168 (39.0) | 129 (50.2) | <0.001 |
| Married/Cohabiting | 752 (68.4) | 263 (61.0) | 128 (49.8) |  |
| Smoking status |  |  |  |  |
| Non-smoking | 467 (42.5) | 234 (54.3) | 147 (57.2) | <0.001 |
| Current smoking | 633 (57.6) | 197 (45.7) | 110 (42.8) |  |
| Alcohol consumption |  |  |  |  |
| Non-drinkers | 492 (44.7) | 273 (63.3) | 203 (79.0) | <0.001 |
| Moderate drinkers | 414 (37.6) | 113 (26.2) | 31 (12.1) |  |
| Heavy drinkers | 194 (17.6) | 45 (10.4) | 23 (9.0) |  |
| MVPA at least 3 times a week | | |  |  |
| No | 369 (33.6) | 247 (57.3) | 213 (82.9) | <0.001 |
| Yes | 731 (66.5) | 184 (42.7) | 44 (17.1) |  |
| BMI (kg/m²) |  |  |  |  |
| <25 | 324 (29.5) | 123 (28.5) | 56 (21.8) | <0.001 |
| 25-30 | 436 (39.6) | 153 (35.5) | 72 (28.0) |  |
| >30 | 340 (30.9) | 155 (36.0) | 129 (50.2) |  |
| Number of comorbidities |  |  |  |  |
| 0 | 202 (18.4) | 26 (6.0) | 9 (3.5) | <0.001 |
| 1 | 326 (29.6) | 68 (15.8) | 21 (8.2) |  |
| 2 | 260 (23.6) | 100 (23.2) | 40 (15.6) |  |
| 3 or more | 312 (28.4) | 237 (55) | 187 (72.8) |  |
| **ADL limitation Score (0-7)** **^ǂ^** |  |  |  |  |
| Mean (SD) | 0.00 (0.00) | 0.93 (0.90) | 2.66 (1.66) | <0.001 |
| **IADL limitation Score (0-6) ^ǂ^** |  |  |  |  |
| Mean (SD) | 0.00 (0.00) | 0.86 (1.08) | 1.37 (1.57) | <0.001 |

*Interview maximum 2 years before stroke. †Values are numbers (percentages). Percentages are reported in column. ǂScore corresponds to number of ADL or IADL limitations. Abbreviations: ADL, Activities of daily living. IADL, Instrumental ADL. P-mRS, premorbid modified Ranking Scale. MVPA, moderate-to-vigorous physical activity.

**Table S2. Characteristics of the study sample aged ≥75 at last* interview before stroke onset according premorbid disability level using modified Rankin scale (P-mRs)†.**

|  | **Premorbid Disability Level of participants aged ≥75** | | | ***p*** |
| --- | --- | --- | --- | --- |
| **Characteristics** | **P-mRs 0-1** | **P-mRs 2-3** | **P-mRs 4-5** |  |
|  | (N=772) | (N= 494) | (N=378) |  |
| Sex |  |  |  |  |
| Men | 390 (50.5) | 165 (33.4) | 109 (28.8) | <0.001 |
| Women | 382 (49.5) | 329 (66.6) | 269 (71.2) |  |
| Age (years) |  |  |  |  |
| 75-81 | 457 (59.2) | 232 (47.0) | 161 (42.6) | <0.001 |
| ≥82 | 315 (40.8) | 262 (53.0) | 217 (57.4) |  |
| Education level |  |  |  |  |
| Low | 257 (33.3) | 204 (41.3) | 206 (54.5) | <0.001 |
| Middle | 351 (45.5) | 212 (42.9) | 131 (34.7) |  |
| High | 164 (21.2) | 78 (15.8) | 41 (10.9) |  |
| Marital status |  |  |  |  |
| Single/divorced/widowed | 347 (45.0) | 244 (49.4) | 220 (58.2) | <0.001 |
| Married/Cohabiting | 425 (55.1) | 250 (50.6) | 158 (41.8) |  |
| Smoking status |  |  |  |  |
| Non-smoking | 422 (54.7) | 344 (69.6) | 257 (68.0) | <0.001 |
| Current smoking | 350 (45.3) | 150 (30.4) | 121 (32.0) |  |
| Alcohol consumption |  |  |  |  |
| Non-drinkers | 408 (52.9) | 313 (63.4) | 303 (80.2) | <0.001 |
| Moderate drinkers | 222 (28.8) | 95 (19.2) | 38 (10.1) |  |
| Heavy drinkers | 142 (18.4) | 86 (17.4) | 37 (9.8) |  |
| MVPA at least 3 times a week | | |  |  |
| No | 331 (42.9) | 317 (64.2) | 331 (87.6) | <0.001 |
| Yes | 441 (57.1) | 177 (35.8) | 47 (12.4) |  |
| BMI (kg/m²) |  |  |  |  |
| <25 | 284 (36.8) | 166 (33.6) | 111 (29.4) | <0.001 |
| 25-30 | 343 (44.4) | 201 (40.7) | 143 (37.8) |  |
| >30 | 145 (18.8) | 127 (25.7) | 124 (32.8) |  |
| Number of comorbidities |  |  |  |  |
| 0 | 120 (15.5) | 24 (4.9) | 16 (4.2) | <0.001 |
| 1 | 241 (31.2) | 98 (19.8) | 45 (11.9) |  |
| 2 | 189 (24.5) | 143 (29.0) | 73 (19.3) |  |
| 3 or more | 222 (28.8) | 229 (46.4) | 244 (64.6) |  |
| **ADL limitation Score (0-7)^ǂ^** | |  |  |  |
| Mean (SD) | 0.00 (0.00) | 0.98 (0.96) | 2.91 (1.85) | <0.001 |
| **IADL limitation Score (0-6)^ǂ^** | |  |  |  |
| Mean (SD) | 0.00 (0.00) | 1.08 (1.36) | 1.98 (2.02) | <0.001 |

*Interview maximum 2 years before stroke. †Values are numbers (percentages). Percentages are reported in column. ǂScore corresponds to number of ADL or IADL limitations. Abbreviations: ADL, Activities of daily living. IADL, Instrumental ADL. P-mRS, premorbid modified Ranking Scale. MVPA, moderate-to-vigorous physical activity.

**Table S3. Odds Ratio of premorbid disability status (P-mRs ≥2) at last* interview before stroke onset by sociodemographic factor, health behaviours and comorbidities**†**.**

| **Participant aged 50-74** | | |  | **Participant aged ≥75** | | |
| --- | --- | --- | --- | --- | --- | --- |
| **Premorbid disability** | **OR (95% CI)** | ***p*** |  | **Premorbid disability** | **OR (95% CI)** | ***p*** |
| Sex |  |  |  | Sex |  |  |
| Men | Ref. | - |  | Men | Ref. | - |
| Women | 1.24 (1.00-1.54) | 0.049 |  | Women | 1.90 (1.50-2.41) | <0.001 |
| Education level |  |  |  | Education level |  |  |
| Low | 1.75 (1.29-2.38) | <0.001 |  | Low | 1.90 (1.40-2.59) | <0.001 |
| Middle | 1.06 (0.79-1.41) | 0.714 |  | Middle | 1.11 (0.82-1.51) | 0.502 |
| High | Ref. | - |  | High | Ref. | - |
| Marital status |  |  |  | Marital status |  |  |
| Single/divorced/widowed | 1.34 (1.08-1.67) | 0.008 |  | Single/divorced/widowed | 1.21 (0.95-1.53) | 0.116 |
| Married/Cohabiting | Ref. | - |  | Married/Cohabiting | Ref. | - |
| BMI |  |  |  | BMI |  |  |
| <25 | Ref. | - |  | <25 | Ref. | - |
| 25-30 | 0.91 (0.70-1.19) | 0.493 |  | 25-30 | 1.12 (0.87-1.43) | 0.362 |
| >30 | 1.12 (0.86-1.46) | 0.388 |  | >30 | 1.75 (1.31-2.35) | <0.001 |
| Number of comorbidities |  |  |  | Number of comorbidities |  |  |
| 0 | Ref. | - |  | 0 | Ref. | - |
| 1 | 1.49 (0.96-2.31) | 0.070 |  | 1 | 1.73 (1.12-2.66) | 0.013 |
| 2 | 2.81 (1.85-4.29) | <0.001 |  | 2 | 3.05 (1.99-4.67) | <0.001 |
| 3 | 6.73 (4.52-10.02) | <0.001 |  | 3 | 5.70 (3.78-8.60) | <0.001 |
| Age of stroke onset |  |  |  | Age of stroke onset |  |  |
| 1-year of increment | 1.01 (0.99-1.02) | 0.428 |  | 1-year of increment | 1.09 (1.06-1.11) | <0.001 |

*Interview maximum 2 years before stroke. †result of multivariate logistic model; OR: Odds Ratio, CI: Confidence Interval

**Table S4. Means of ADL limitation score in stroke survivors by premorbid modified Rankin scale (P-mRS) stratified by age*.**

|  | **Means of ADL limitation score (95% Confidence Interval)** | | | | | | |
| --- | --- | --- | --- | --- | --- | --- | --- |
| **Years follow up** | **aged at 50 - 74** | | |  | **aged at 75 and over** | | |
|  | **P-mRS 0-1** | **P-mRS 2-3** | **P-mRS 4-5** |  | **P-mRS 0-1** | **P-mRS 2-3** | **P-mRS 4-5** |
| Pre-stroke | 0.10 (0.03-0.17) | 0.93 (0.83-1.03) | 2.58 (2.45-2.71) |  | 0.11 (0.01-0.22) | 0.99 (0.87-1.11) | 2.83 (2.69-2.96) |
| 1 | 0.74 (0.65-0.84) | 1.24 (1.11-1.37) | 2.62 (2.46-2.79) |  | 1.22 (1.07-1.36) | 2.20 (2.03-2.37) | 3.59 (3.40-3.79) |
| 2 | 0.72 (0.64-0.80) | 1.30 (1.18-1.41) | 2.62 (2.47-2.77) |  | 1.32 (1.19-1.46) | 2.24 (2.08-2.40) | 3.59 (3.40-3.78) |
| 3 | 0.72 (0.64-0.80) | 1.35 (1.23-1.48) | 2.64 (2.47-2.8) |  | 1.48 (1.32-1.64) | 2.33 (2.13-2.52) | 3.65 (3.42-3.89) |
| 4 | 0.73 (0.63-0.83) | 1.41 (1.26-1.56) | 2.67 (2.47-2.87) |  | 1.68 (1.48-1.88) | 2.45 (2.20-2.69) | 3.78 (3.49-4.08) |
| 5 | 0.76 (0.64-0.88) | 1.47 (1.30-1.65) | 2.72 (2.49-2.96) |  | 1.93 (1.69-2.17) | 2.60 (2.30-2.90) | 3.98 (3.61-4.34) |
| 6 | 0.80 (0.67-0.94) | 1.53 (1.33-1.73) | 2.79 (2.52-3.06) |  | 2.22 (1.93-2.51) | 2.80 (2.44-3.16) | 4.24 (3.80-4.68) |
| 7 | 0.86 (0.71-1.02) | 1.58 (1.35-1.81) | 2.88 (2.57-3.19) |  | 2.56 (2.20-2.92) | 3.03 (2.59-3.46) | 4.57 (4.03-5.10) |
| 8 | 0.94 (0.75-1.12) | 1.64 (1.38-1.90) | 2.99 (2.63-3.34) |  | 2.95 (2.50-3.39) | 3.30 (2.76-3.83) | 4.96 (4.30-5.62) |
| 9 | 1.03 (0.81-1.24) | 1.69 (1.40-1.99) | 3.11 (2.71-3.51) |  | 3.38 (2.82-3.93) | 3.60 (2.94-4.27) | 5.42 (4.61-6.24) |
| 10 | 1.13 (0.88-1.38) | 1.75 (1.41-2.09) | 3.25 (2.80-3.71) |  | 3.86 (3.16-4.55) | 3.94 (3.12-4.77) | 5.95 (4.94-6.95) |
| 11 | 1.25 (0.95-1.55) | 1.80 (1.41-2.20) | 3.42 (2.90-3.93) |  | 4.38 (3.52-5.24) | 4.32 (3.30-5.34) | 6.54 (5.31-7.78) |
| 12 | 1.39 (1.03-1.75) | 1.85 (1.40-2.31) | 3.59 (3.01-4.18) |  | 4.95 (3.89-6.01) | 4.74 (3.50-5.98) | 7.20 (5.70-8.70) |
| 13 | 1.54 (1.11-1.97) | 1.91 (1.38-2.44) | 3.79 (3.12-4.46) |  | - | - | - |
| 14 | 1.71 (1.20-2.22) | 1.96 (1.34-2.58) | 4.01 (3.24-4.77) |  | - | - | - |
| 15 | 1.89 (1.30-2.49) | 2.01 (1.29-2.72) | 4.24 (3.37-5.12) |  | - | - | - |
| 16 | 2.09 (1.39-2.79) | 2.06 (1.23-2.88) | 4.49 (3.49-5.49) |  | - | - | - |

*Estimated from piecewise linear mixed model adjusted for socioeconomic variables, pre-stroke wave data collection, heath behaviours, BMI, and number of comorbidities. ADL score range from 0=no limitation to 7=maximum limitation. Estimations are presented for a 16-year follow-up (corresponding to maximum follow-up with at least 5 participants by level of mRS) for population aged 50-74 years and a 12-year follow-up for those aged ≥75 years. Abbreviations: ADL, Activities of daily living. IADL, Instrumental ADL. P-mRS, premorbid modified Ranking Scale.

**Table S5. Means of IADL limitation score in stroke survivors by premorbid modified Rankin scale (P-mRS) stratified by age*.**

|  | **Means of IADL limitation score (95% Confidence Interval)** | | | | | | |
| --- | --- | --- | --- | --- | --- | --- | --- |
| **Years follow up** | **aged at 50 - 74** | | |  | **aged at 75 and over** | | |
|  | **P-mRS 0-1** | **P-mRS 2-3** | **P-mRS 4-5** |  | **P-mRS 0-1** | **P-mRS 2-3** | **P-mRS 4-5** |
| Pre-stroke | 0.08 (0.01-0.15) | 0.86 (0.77-0.96) | 1.30 (1.17-1.43) |  | 0.02 (-0.26-0.30) | 1.10 (0.82-1.39) | 1.86 (1.56-2.15) |
| 1 | 0.55 (0.47-0.64) | 1.19 (1.07-1.31) | 1.63 (1.47-1.78) |  | 0.97 (0.67-1.27) | 2.01 (1.70-2.32) | 2.57 (2.25-2.89) |
| 2 | 0.53 (0.46-0.60) | 1.17 (1.06-1.28) | 1.60 (1.46-1.74) |  | 1.09 (0.80-1.38) | 2.03 (1.73-2.33) | 2.69 (2.38-3.00) |
| 3 | 0.53 (0.45-0.60) | 1.17 (1.05-1.28) | 1.59 (1.44-1.74) |  | 1.23 (0.94-1.53) | 2.11 (1.80-2.43) | 2.84 (2.51-3.18) |
| 4 | 0.54 (0.45-0.63) | 1.18 (1.05-1.31) | 1.61 (1.43-1.78) |  | 1.41 (1.09-1.72) | 2.25 (1.91-2.59) | 3.03 (2.66-3.40) |
| 5 | 0.57 (0.46-0.67) | 1.21 (1.05-1.36) | 1.64 (1.44-1.85) |  | 1.61 (1.28-1.95) | 2.44 (2.07-2.81) | 3.24 (2.83-3.66) |
| 6 | 0.61 (0.49-0.73) | 1.25 (1.07-1.43) | 1.70 (1.46-1.94) |  | 1.85 (1.48-2.22) | 2.69 (2.28-3.10) | 3.50 (3.03-3.96) |
| 7 | 0.67 (0.53-0.81) | 1.30 (1.10-1.50) | 1.78 (1.51-2.06) |  | 2.11 (1.70-2.52) | 2.99 (2.53-3.45) | 3.78 (3.24-4.32) |
| 8 | 0.75 (0.59-0.91) | 1.37 (1.14-1.60) | 1.89 (1.58-2.20) |  | 2.41 (1.93-2.88) | 3.35 (2.81-3.89) | 4.10 (3.46-4.74) |
| 9 | 0.84 (0.65-1.03) | 1.45 (1.19-1.71) | 2.01 (1.66-2.36) |  | 2.73 (2.16-3.30) | 3.77 (3.12-4.41) | 4.45 (3.68-5.22) |
| 10 | 0.95 (0.73-1.18) | 1.55 (1.24-1.85) | 2.16 (1.76-2.56) |  | 3.08 (2.40-3.77) | 4.24 (3.45-5.02) | 4.83 (3.89-5.77) |
| 11 | 1.08 (0.81-1.35) | 1.65 (1.31-2.00) | 2.33 (1.87-2.78) |  | 3.47 (2.63-4.30) | 4.76 (3.80-5.73) | 5.25 (4.10-6.40) |
| 12 | 1.22 (0.90-1.55) | 1.78 (1.37-2.18) | 2.52 (2.00-3.04) |  | 3.88 (2.87-4.89) | 5.35 (4.18-6.52) | 5.70 (4.30-7.10) |
| 13 | 1.38 (1.00-1.77) | 1.92 (1.44-2.39) | 2.73 (2.14-3.33) |  | - | - | - |
| 14 | 1.56 (1.10-2.02) | 2.07 (1.52-2.62) | 2.97 (2.28-3.65) |  | - | - | - |
| 15 | 1.75 (1.21-2.29) | 2.23 (1.59-2.88) | 3.22 (2.44-4.01) |  | - | - | - |
| 16 | 1.96 (1.33-2.59) | 2.41 (1.67-3.16) | 3.50 (2.60-4.40) |  |  |  |  |

*Estimated from piecewise linear mixed model adjusted for socioeconomic variables, pre-stroke wave data collection, heath behaviours, BMI, and number of comorbidities. IADL score range from 0=no limitation to 6=maximum limitation. Estimations are presented for a 16-year follow-up (corresponding to maximum follow-up with at least 5 participants by level of mRS) for population aged 50-74 years and a 12-year follow-up for those aged ≥75 years. Abbreviations: ADL, Activities of daily living. IADL, Instrumental ADL. P-mRS, premorbid modified Ranking Scale.

**Table S6. Mortality report during post-stroke follow-up by premorbid modified Rankin scale (P-mRS)*.**

| **Year of mortality report** | **Premorbid disability level** | | |
| --- | --- | --- | --- |
|  | **P-mRS 0-1 (%)** | **P-mRS 2-3 (%)** | **P-mRS 4-5 (%)** |
| 1-3 | 49 (23.0) | 65 (32.5) | 81 (40.9) |
| 4-6 | 79 (37.1) | 63 (31.5) | 61 (30.8) |
| 7-9 | 42 (19.7) | 41 (20.5) | 35 (17.7) |
| 10-12 | 25 (11.7) | 18 (9.0) | 13 (6.6) |
| 13-15 | 12 (5.6) | 11 (5.5) | 6 (3.0) |
| ≥16 | 6 (2.8) | 2 (1.0) | 2 (1.0) |
| Total | 213 (100) | 200 (100) | 198 (100) |

*Percentage by column


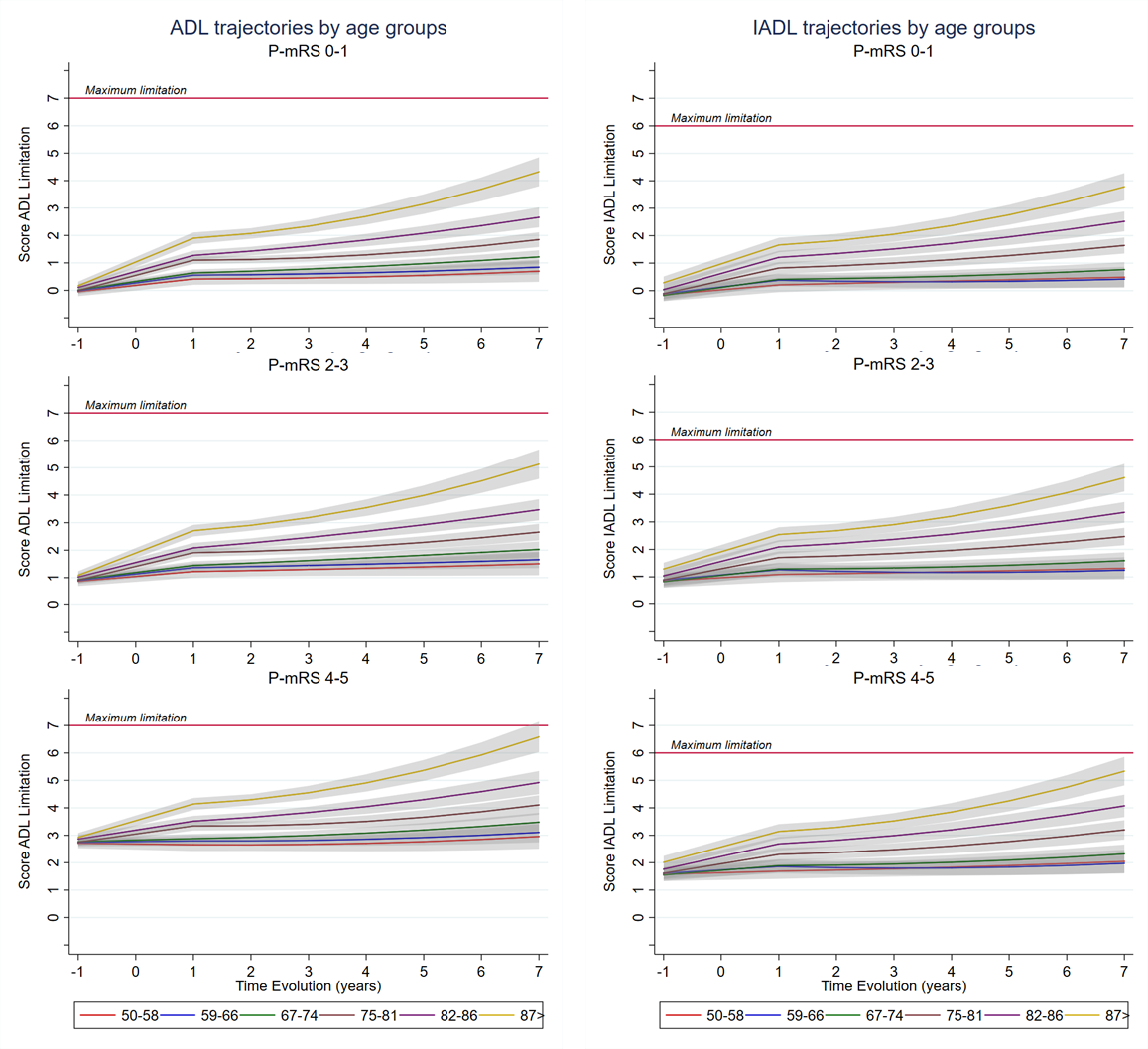
**Figure S1. Long-term limitations of score of ADL/IADL in stroke survivors by age groups and premorbid modified Rankin scale (P-mRS)*.**

*Estimated from piecewise linear mixed model adjusted for sex and education. Breakpoint was set at 1-year post-stroke. .Detailed changes in limitations between pre-stroke and 1-year post-stroke could not be modelled due to lack of information and was assumed linear for the sake of the analysis. ADL score range from 0=no limitation to 7=maximum limitation.

**Figure S2. Long-term trajectories of score of ADL limitations in stroke survivors by premorbid modified Rankin scale (P-mRS) stratified by age and survey*.**

**
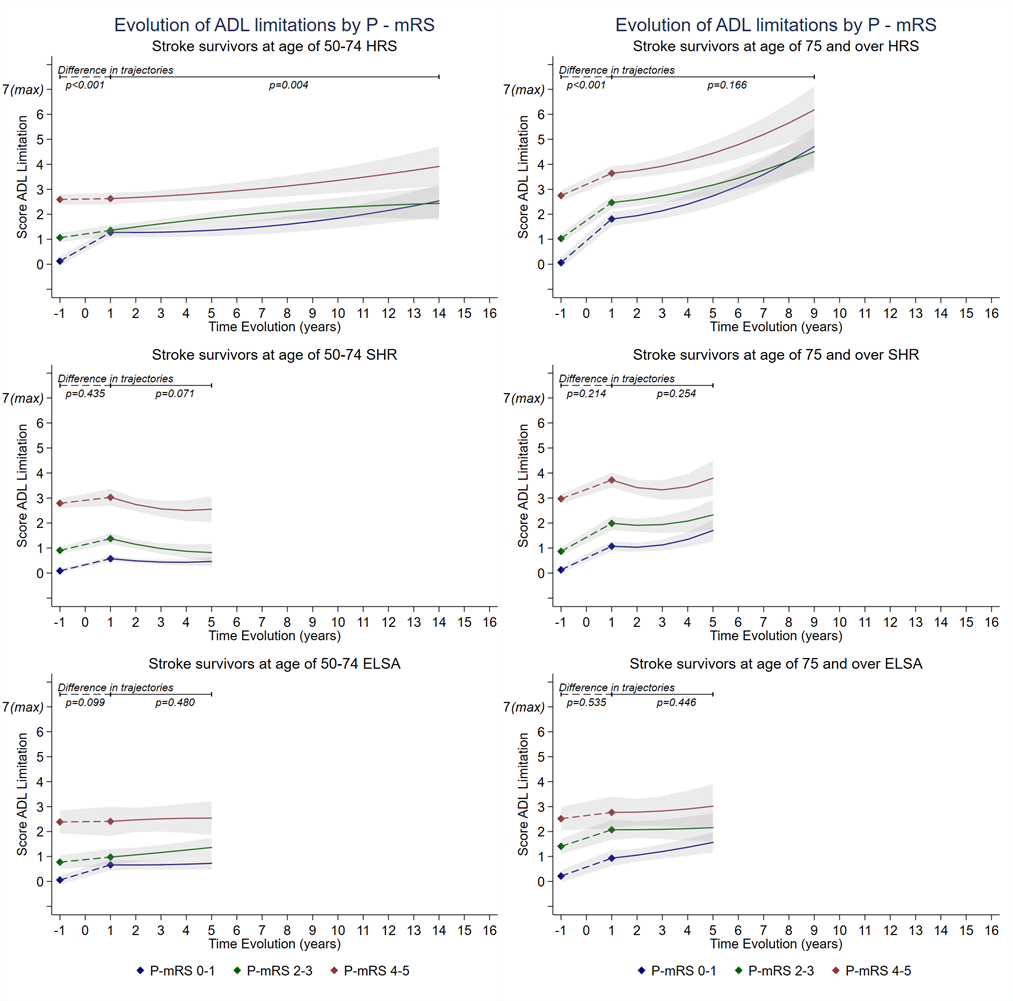
**

*Estimated from piecewise linear mixed model adjusted for socioeconomic variables, pre-stroke wave data collection, heath behaviours, BMI, and number of comorbidities. Breakpoint was set at 1-year post-stroke. Detailed changes in limitations between pre-stroke and 1-year post-stroke could not be modelled due to lack of information and was assumed linear for the sake of the analysis. ADL score range from 0=no limitation to 7=maximum limitation.

**Figure S3. Long-term trajectories of score of IADL limitations in stroke survivors by premorbid modified Rankin scale (P-mRS) stratified by age and survey*.**

**
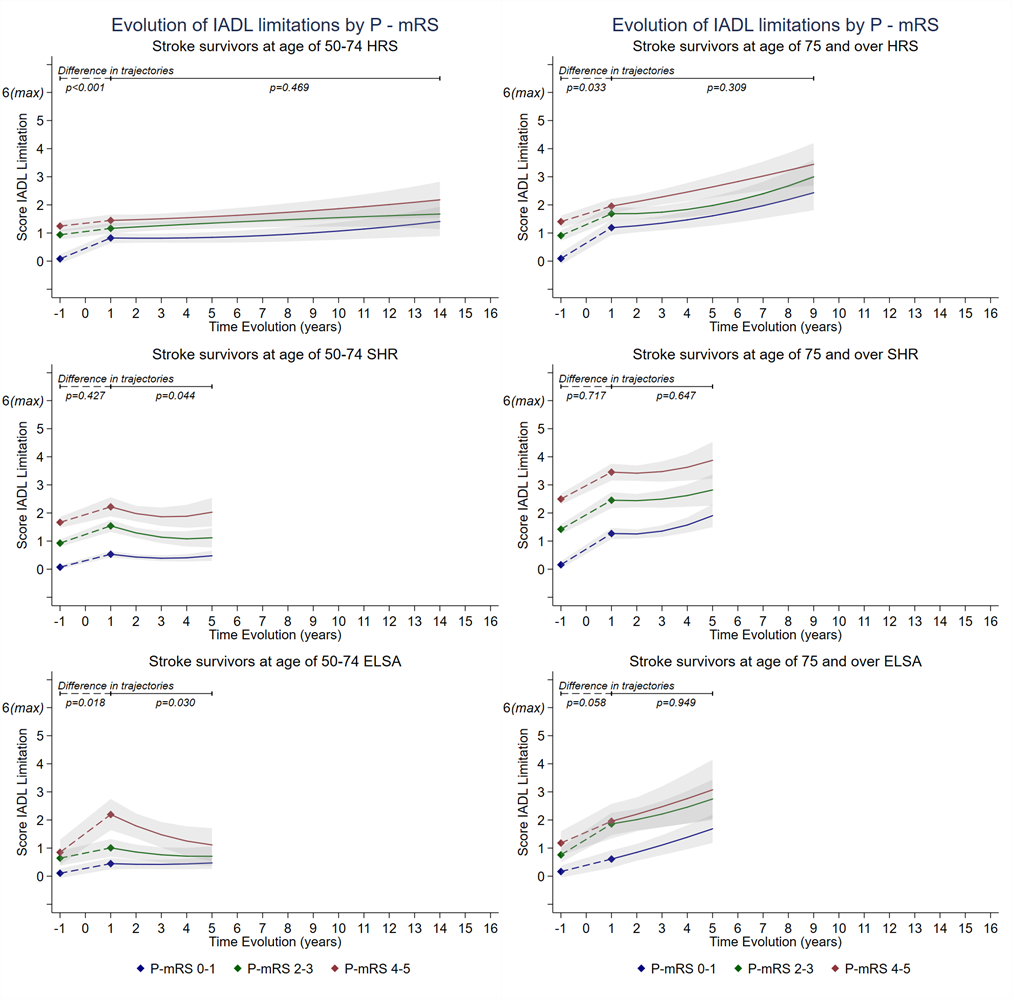
**

*Estimated from piecewise linear mixed model adjusted for socioeconomic variables, pre-stroke wave data collection, heath behaviours, BMI, and number of comorbidities. Breakpoint was set at 1-year post-stroke. Detailed changes in limitations between pre-stroke and 1-year post-stroke could not be modelled due to lack of information and was assumed linear for the sake of the analysis. IADL score range from 0=no limitation to 6=maximum limitation.

**Figure S4. Long-term trajectories of score of ADL/IADL limitations in stroke survivors by premorbid modified Rankin scale (P-mRS), stratified by age and with partial imputation at 1-year pre-stroke*.**


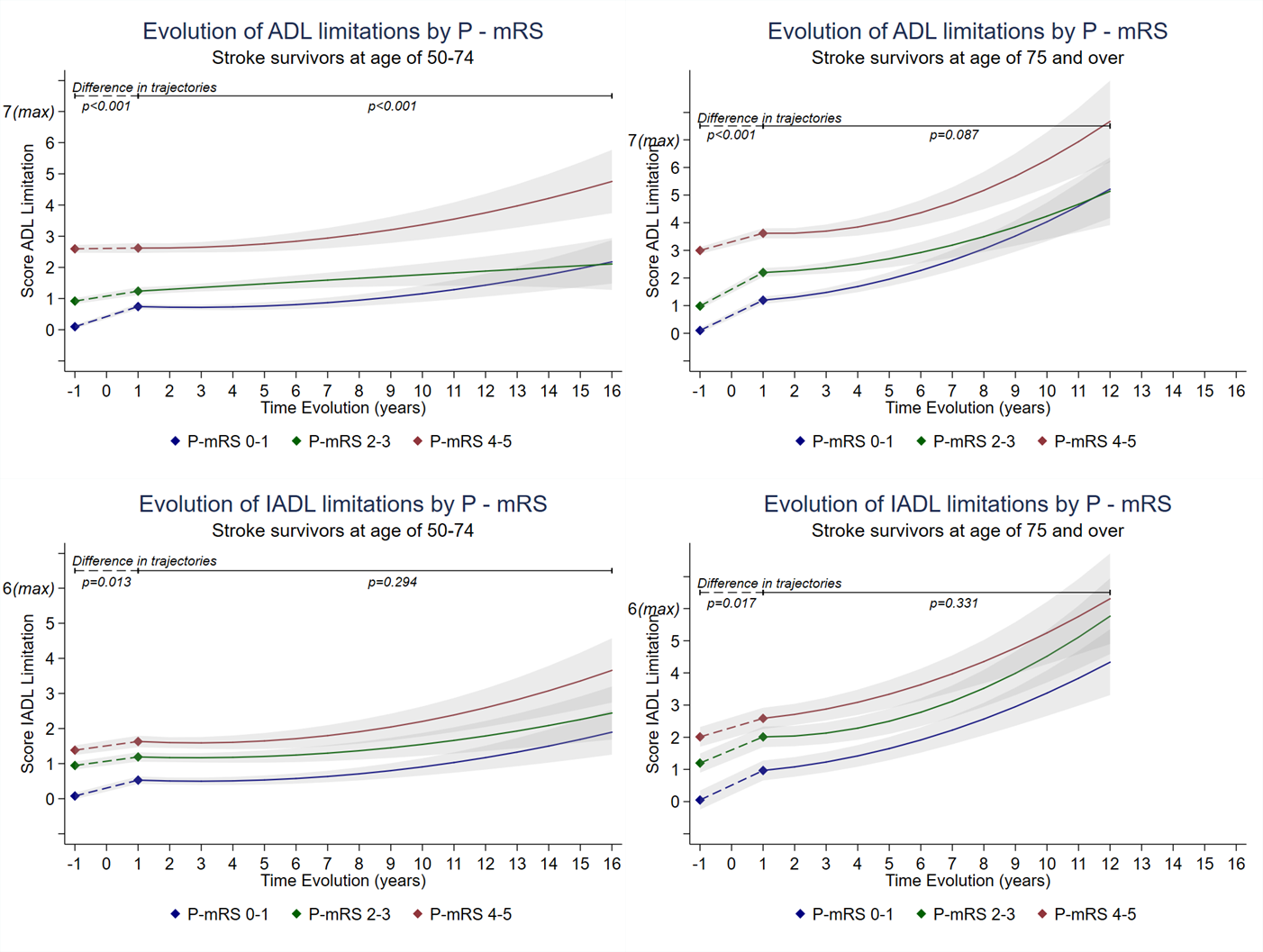


*Estimated from piecewise linear mixed model adjusted for socioeconomic variables, pre-stroke wave data collection, heath behaviours, BMI, and number of comorbidities. Breakpoint was set at 1-year post-stroke. Detailed changes in limitations between pre-stroke and 1-year post-stroke could not be modelled due to lack of information and was assumed linear for the sake of the analysis. ADL/IADL score range from 0=no limitation to 7/6=maximum limitation. Estimations are presented for a 16-year follow-up (corresponding to maximum follow-up with at least 5 participants by level of mRS) for population aged 50-74 years and a 12-year follow-up for those aged ≥75 years.

**Figure S5. Long-term trajectories of score of ADL/IADL limitations in stroke survivors by premorbid modified Rankin scale (P-mRS) stratified by age and follow-up limited to 6 years post-stroke*.**

**
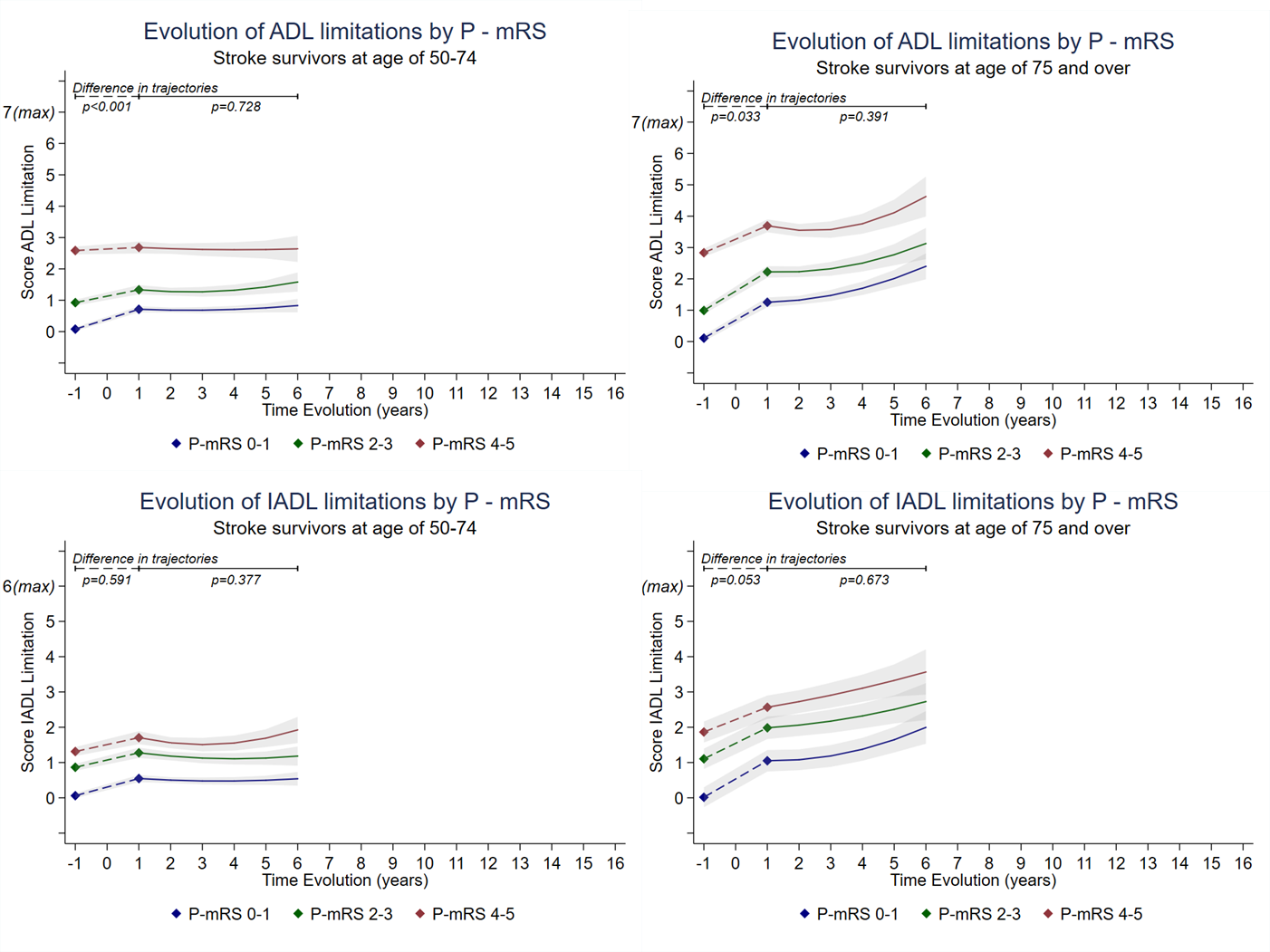
**

*Estimated from piecewise linear mixed model adjusted for socioeconomic variables, pre-stroke wave data collection, heath behaviours, BMI, and number of comorbidities. Breakpoint was set at 1y-ear post-stroke. Detailed changes in limitations between pro-stroke and 1-year post-stroke could not be modelled due to lack of information and was assumed linear for the sake of the analysis. ADL/IADL score range from 0=no limitation to 7/6=maximum limitation.

**Figure S6. Long-term trajectories of score of ADL/IADL limitations in stroke survivors by premorbid modified Rankin scale (P-mRS) stratified by age and follow-up excluding participants with report of dead*.**

**
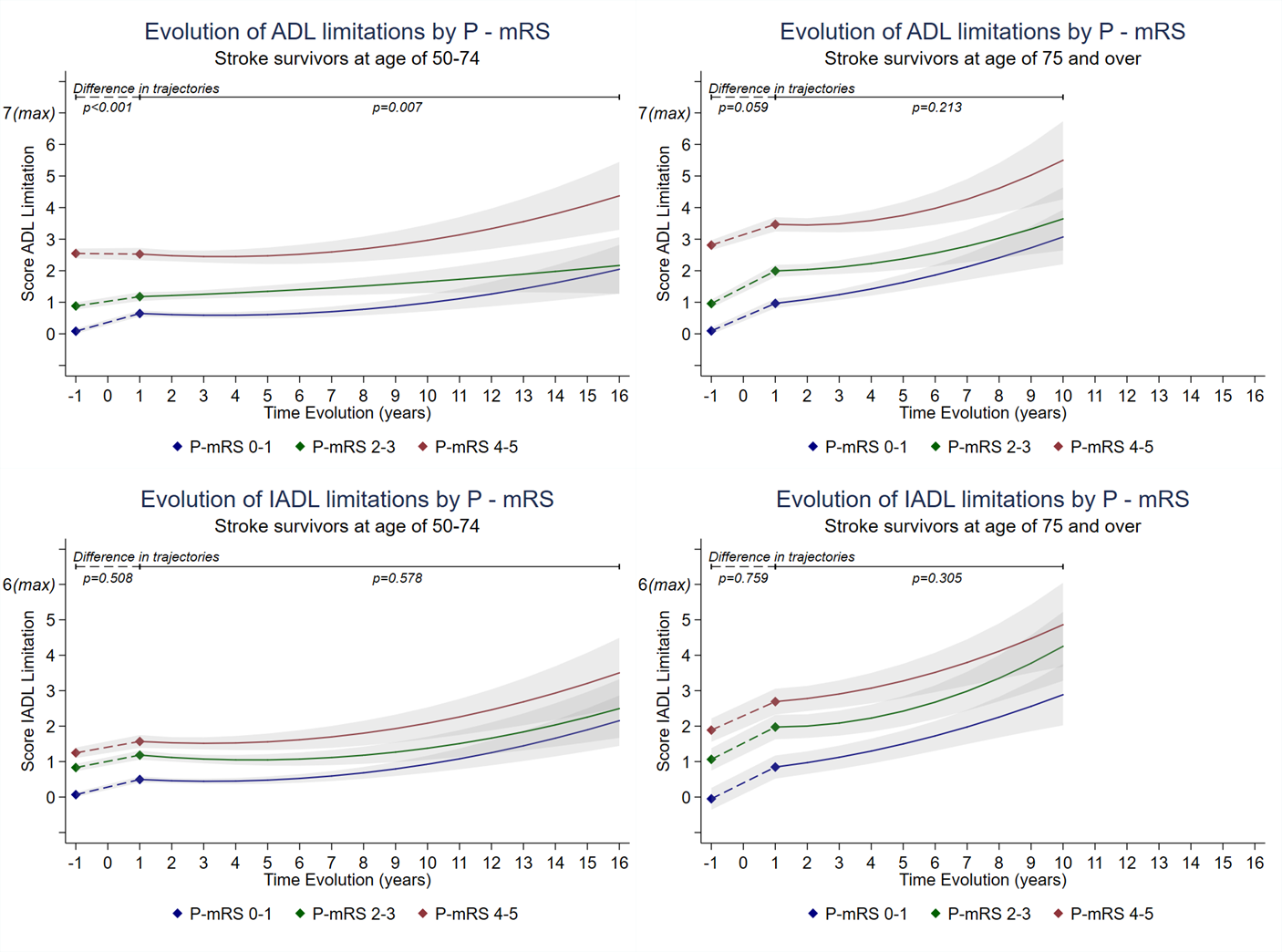
**

*Estimated from piecewise linear mixed model adjusted for socioeconomic variables, pre-stroke wave data collection, heath behaviours, BMI, and number of comorbidities. Breakpoint was set at 1-year post-stroke. Detailed changes in limitations between pro-stroke and 1-year post-stroke could not be modelled due to lack of information and was assumed linear for the sake of the analysis. ADL/IADL score range from 0=no limitation to 7/6=maximum limitation. Estimations are presented for a 16-year follow-up (corresponding to maximum follow-up with at least 5 participants by level of mRS) for population aged 50-74 years and a 10-year follow-up for those aged ≥75 years.
